# *Notes from the Field:* Six Cases of Acute Flaccid Myelitis in Children — Minnesota, 2018

**DOI:** 10.15585/mmwr.mm6815a4

**Published:** 2019-04-19

**Authors:** Heidi Moline, Anupama Kalaskar, William F. Pomputius, Adriana Lopez, Janell Routh, Cynthia Kenyon, Jayne Griffith

**Affiliations:** ^1^Department of Pediatrics, University of Minnesota Masonic Children’s Hospital, University of Minnesota Medical School, Minneapolis, Minnesota; ^2^Division of Infectious Disease, Children’s Hospitals and Clinics of Minnesota, Minneapolis, Minnesota; ^3^National Center for Immunization and Respiratory Diseases, CDC; ^4^Infectious Disease Epidemiology, Prevention and Control Division, Minnesota Department of Health.

During September 14–October 1, 2018, the Minnesota Department of Health (MDH) was notified of six children hospitalized in the Minneapolis-St. Paul region with symptoms consistent with acute flaccid myelitis (AFM). A confirmed case of AFM is defined as acute onset of flaccid limb weakness with magnetic resonance image indicating spinal cord lesions largely restricted to gray matter and spanning one or more vertebral segments (*1*). All six cases were confirmed by CDC. After a cluster of three cases occurred in 2014, an average of fewer than one AFM case per year had been reported to MDH.

Among the six patients, the median patient age was 6.0 years (range = 1.3–9.2 years). All children resided in different Minnesota counties, and all experienced fever and upper respiratory signs and symptoms (e.g., rhinorrhea and cough) beginning a median of 8 days (range = 5–11 days) before weakness onset; none had a history of being immunocompromised. In addition, four patients experienced neck pain or headache, and two experienced diarrhea before weakness onset. Four patients had marked weakness of proximal muscle groups in one arm, although distal motor function was largely preserved. The other two patients initially had weakness in one leg, which became bilateral and rapidly ascended during hospitalization; both of these patients required endotracheal intubation and mechanical ventilation. In all six patients, limb weakness was first noted after waking in the morning. No epidemiologic links among patients were identified.

All six patients were hospitalized. Three patients were discharged home, and two were discharged to inpatient rehabilitation facilities. One patient remains hospitalized with complete paralysis of all voluntary muscles, including the diaphragm, at the time of this report. All discharged patients had residual weakness at time of discharge; among these patients, the median duration of hospitalization was 8 days (range = 1–14 days).

Magnetic resonance imaging (MRI) indicated spinal cord gray matter involvement in all six patients, largely in the anterior horns. The extent of gray matter involvement did not always correlate with deficits seen on physical exam; in three patients with only single limb weakness, multisegment gray matter involvement was apparent. Among all patients, three had anterior nerve root and facial nerve enhancement, and two had basilar and brainstem involvement. Three patients had normal MRI findings early in the illness course, but demonstrated extensive gray matter involvement on a subsequent MRI.

Cerebrospinal fluid (CSF) was collected in five patients, with pleocytosis (white blood cell count >5 cells/mm^3^) present in two patients (Table). One CSF specimen (patient B) was positive for enterovirus (not typed) by reverse transcription–polymerase chain reaction (RT-PCR) at a commercial reference laboratory. Serum, CSF, stool, and nasopharyngeal specimens from five patients were tested at CDC. One nasopharyngeal swab (patient D) was positive for enterovirus-D68 (EV-D68) by real-time RT-PCR. One nasal wash specimen from patient B was positive for EV-D68 and a second specimen for EV-D68 and parechovirus A6 by real-time RT-PCR; CSF from this patient also was positive for EV-D68. The remaining specimens were negative, including those from three patients who had no positive specimens. All stool specimens were negative for poliovirus.

**TABLE:** Demographic characteristics, clinical findings and evaluation, hospital course, and outcome among six patients with acute flaccid myelitis — Minnesota, September–October 2018

Characteristic	Patient A	Patient B	Patient C	Patient D	Patient E	Patient F
Age	7 yrs	7 yrs	16 mos	3 yrs	9 yrs	5 yrs
Sex	Male	Female	Female	Female	Female	Female
Previous/Underlying medical conditions	None	None	Cerebral palsy, seizure disorder	Congenital cataract	None	None
Viral prodrome period	Sep 9–11	Sep 9–13	Sep 17–19	Sep 16–18	Sep 17–21	Sep 21–26
Other symptoms preceding weakness onset	Headache, vomiting, body aches	Headache	Diarrhea	Headache, neck ache, vomiting, diarrhea	None	Neck ache
Weakness onset date	Sep 14	Sep 19	Sep 22	Sep 23	Sep 24	Sep 29
Weakness site	Left arm	Left leg	Left leg	Left arm	Right arm	Right arm
Hospital admission date	Sep 20	Sep 19	Sep 22	Sep 25	Sep 28	Oct 1
Magnetic resonance Imaging findings	HD 1: Normal	HD 1: Enhancement of meninges; gray matter in thoracic cord	HD 1: Normal	HD 1: Normal	HD 1: Enhancement of gray matter in cervical and thoracic cord	HD 2: Enhancement of cervical and brainstem gray matter
	HD 7: Enhancement of cervical and brainstem anterior horn, cauda equina	HD 8: Improved thoracic cord enhancement; new cervical, cauda equina, and frontal lobe enhancement	HD 3: Enhancement of gray matter from cervical cord to cauda equine	HD 3: Extensive enhancement of cervical and thoracic anterior horn		
Cerebrospinal fluid test results	HD 1: No pleocytosis; no viral detection	HD 1: Pleocytosis; no virus detected	HD 1: Pleocytosis; no virus detected	HD 1: No pleocytosis; no virus detected	Not collected	HD 1: No pleocytosis; no virus detected
		HD 3: Pleocytosis; EV-D68 positive				
		HD 9: Pleocytosis; no virus detected				
Nasopharyngeal swab test results	HD 7: No virus detected	HD 3: EV-D68 positive	HD 1: No virus detected	HD 1: EV-D68 positive	Not collected	HD 1: No virus detected
		HD 10: EV-D68 positive; PEV-A6 positive				
Treatment	Steroids, IVIG	Plasmapheresis, steroids, IVIG	IVIG	IVIG	None	IVIG
Hospital course	Left arm and left facial weakness noted at admission; facial weakness improved; arm weakness with minimal improvement at discharge	Rapidly ascending paralysis; respiratory failure; loss of all voluntary motor function; pupillary response intact; cognitively intact; no clinical improvement	Ascending paralysis; respiratory failure; gradual improvement of weakness; persistent left leg weakness and dysphagia at discharge	Left arm and left facial weakness at admission; resolution of facial weakness; improved arm weakness at discharge	Right arm weakness at admission; mild improvement of weakness at discharge	Right arm and neck weakness at admission; improvement in neck weakness; minimal improvement of arm weakness at discharge
Discharge date	Oct 3	Not applicable	Oct 4	Oct 3	Sep 29	Oct 10
No. of days hospitalized	14	>90 (ongoing)	12	9	1	9
Discharge location	Home	Not applicable	Inpatient rehabilitation	Home	Home	Inpatient rehabilitation

**Abbreviations:** EV = enterovirus; HD = hospital day; IVIG = intravenous immunoglobulin; PEV = parechovirus.

Five of six patients received some form of immunomodulatory treatment (Table). One patient was treated with steroids and plasmapheresis followed by intravenous immune globulin (IVIG), one with steroids followed by IVIG, three with only IVIG, and one with supportive care only.

This AFM cluster, the largest identified in Minnesota, occurred during a period of increased reporting of AFM nationally and is consistent with the epidemiologic and clinical characteristics of previously described AFM clusters (*2*–*6*). Despite report of upper respiratory tract signs and symptoms in all patients, testing for viruses that commonly cause upper respiratory tract infections was positive from nonsterile specimens in only two cases. EV-D68 in the CSF of patient B is considered the cause of AFM in this patient. Detection of a pathogen in the CSF might be related to the severity and prolonged nature of illness in this patient; however, host or other factors contributing to illness severity are unknown.

AFM is a rare but serious cause of sudden onset limb weakness, especially in children, and should be considered in the differential diagnosis. Diagnosis and care of patients with AFM includes early collection of specimens, including CSF, for laboratory testing, MRI scans, and consultation with neurology and infectious disease experts. Potential cases should be reported to public health departments in a timely manner. Public health classification of AFM cases involves expert review of clinical and imaging findings; however, it is important that clinical care not be delayed pending case classification.
